# Enrichment of *Parachlorella kessleri* biomass with bioproducts: oil and protein by utilization of beet molasses

**DOI:** 10.1007/s10811-017-1081-y

**Published:** 2017-02-13

**Authors:** Agata Piasecka, Izabela Krzemińska, Jerzy Tys

**Affiliations:** Institute of Agrophysics, Polish Academy of Sciences, Doświadczalna 4, 20-290 Lublin, Poland

**Keywords:** Beet molasses, Biodiesel, Calorific value, *Parachlorella kessleri*, Chlorophyta, Proteins

## Abstract

The aim of this study was to determine the suitability of beet molasses, an agro-industrial by-product, as an alternative culture medium component for photoheterotrophic and mixotrophic cultivation of *Parachlorella kessleri*. Application of beet molasses improved microalgal cell growth and modified the biochemical composition of *P. kessleri* biomass. During the addition of molasses to culture media with simultaneous aeration, the maximum biomass productivity, oil and protein productivity, and calorific value were 0.42 g L^−1^ day^−1^, 112.56 and 244.95 mg L^−1^ day^−1^, and 22.1 MJ kg^−1^, respectively. Under these conditions, the total content of polyunsaturated C16-C18 fatty acids decreased, which was suitable for application in biodiesel. Besides oils and carbohydrates, *P. kessleri* had an ability to synthesize significant amounts of proteins, especially during molasses utilization. This provides a possibility of a wide range of non-fuel applications of *P. kessleri* biomass.

## Introduction

Algal biofuels have been considered as one of the most promising options to provide global energy needs. However, algal technology has yet to overcome the cost of production and processing technology. It is important to reduce the cost of microalgal biomass production. Under mixotrophic conditions based on wastewater media, microalgae may bring flexibility to improve production economics while generating valuable products (Lowrey et al. 2015). Organic carbon sources like glucose or acetate are usually costly and are responsible for most of the medium costs. A cheap industrial by-product, such as molasses, could be used as a low-cost medium supplement to reduce the costs of microalgal biomass production. In addition, the technology of microalgal culturing is becoming greener (Yan et al. 2011; Mitra et al. 2012). Molasses is a by-product in a sugar industry consisting of approximately 50% of total sugars, predominantly sucrose, but containing significant amounts of reducing sugars—glucose and fructose and other carbohydrates (Polish Standard PN-R-64772, PN-ISO 6496:2002). The non-sugar content includes many metal ions, such as calcium, magnesium, potassium, sodium, iron, and cooper (Liu et al. 2012). It is also a source of nitrogen. Until now, molasses has been reported as a carbon source for the production of *Botryococcus braunii*, *Scenedesmus obliquus*, *Auxenochlorella protothecoides* (*Chlorella protothecoides*), *Chromochloris zofingiensis* (*Chlorella zofingiensis*), and *Chlorella minutissima* biomass for astaxanthin or biodiesel production (Yan et al. 2011; Liu et al. 2012; El-Sheekh et al. 2013; Gautam et al. 2013; Liu et al. 2013; Yeesang and Cheirsilp 2014).

*Parachlorella kessleri* is a green alga with cells capable of utilizing organic carbon sources such as glucose (Wang et al. 2012), ethanol, and glycerol (Wang et al. 2013). This makes *P. kessleri* a great candidate for photoheterotrophic and mixotrophic cultivation that offers great potential in the production of microalgal renewable biomass for biodiesel production and for different applications. To our knowledge, there is no reported study investigating the use of molasses for *P. kessleri* culturing.

Microalgal biomass production for biodiesel also could be combined with production of other valuable compounds, which may contribute to a direct reduction of costs. Combined production of oil and other bioactive products provides environmental and economic sustainability of microalgal technology (Bellou et al. 2014). The cited authors have reviewed the existing literature and presented polysaccharides, proteins, and pigments as high-value metabolites in combining oil production for commercial applications, especially animal feed, aquaculture (fish feed), and pharmaceutical and nutraceutical products.

The objective of the present study was to investigate the influence of the addition of molasses to the culture medium on *P. kessleri* growth, biomass composition, and calorific value. Additionally, the work was intended to determine the potential of the agro-industrial by-product as an alternative culture medium component that guarantees production of more than one compound from *P. kessleri* biomass.

## Material and methods

### Microalgal strain, culture medium, and inoculum preparation

The initial *Parachlorella kessleri* strain was obtained from the Culture Collection of Autotrophic Organisms (CCALA) at the Charles University in Prague. Kessler medium was used as a basic medium. The Kessler medium (Kessler and Czygan 1970) was prepared on distilled water and was composed of (per litre) 0.81 g KNO_3_, 0.47 g NaCl, 0.47 g NaH_2_PO_4_·2H_2_O, 0.36 g Na_2_HPO_4_·12H_2_O, 0.25 g MgSO_4_·7H_2_O, 0.014 g CaCl_2_·2H_2_O, 0.006 g FeSO_4_·7H_2_O, 0.0005 g MnCl_2_·4H_2_O, 0.0005 g H_3_BO_3_, 0.0002 g ZnSO_4_·7H_2_O, 0.00002 g (NH_4_)_6_Mo_7_O_24_·4H_2_O, and 0.008 g EDTA. In order to prepare the pre-culture, the liquid Kessler medium was inoculated from a 2% agar plate. Pre-culture was incubated in 25 ± 1 °C for 10 days under continuous illumination (80 μmol photons m^−2^ s^−1^) in 50-mL Erlenmeyer flasks and agitated at 100 rpm. The pre-culture was used as an inoculum in the experiment.

### Culture conditions

The effect of the application of beet molasses in culture medium on the growth and biochemical composition of *P. kessleri* biomass was studied. The experimental variants included autotrophic cultivation of (KA), photoheterotrophic cultivation of (KM), and mixotrophic cultivation of (KMA). The control culture/air-limited autotrophic cultivation (K) was prepared by inoculating fresh Kessler medium. This control culture for *P. kessleri* cultivation was not supplied with any carbon source. For autotrophic conditions, the Kessler medium was constantly aerated. Waste beet molasses was obtained from a local sugar refinery (Lublin Province, Poland). To prepare photoheterotrophic and mixotrophic conditions, 10 g L^−1^ molasses was rinsed with demineralised water twice before adding to the Kessler medium and sterilized.

Cultivation was carried out in four variants marked sequentially as K (control), KA (Kessler medium with aeration), KM (Kessler medium with molasses), and KMA (Kessler medium with molasses and aeration). The *P. kessleri* cells were cultivated in 3-L photobioreactors (BIOSTAT PBR 2S Sartorius Stedim Biotech). The cultures were continuously illuminated by fluorescent lamps. The light intensity was 80 μmol photons m^−2^ s^−1^ at 25 ± 1 °C. The KA and KMA cultures were continuously aerated with sterile air at 12 L h^−1^ airflow.

### Growth measurements and biomass determination

Cell density was measured daily using UV/visible spectrophotometry (Cary 300/Biomelt spectrophotometer). Based on the presence of chlorophyll in viable cells, the optical density was measured at 650 nm. The correlation between dry cell weight (DCW, g L^−1^) and optical density (OD_650_) is represented by Eq. 1.

1 $$ \mathrm{DCW}=372.21\times {\mathrm{OD}}_{650}-9.1591,{R}^2=0.9968 $$

The specific growth rate (0–3 days) was calculated using the following formula (Eq. 2) (Krzemińska et al. 2014):

2 $$ \mu \left({d}^{-1}\right)= \ln \left({N}_2/{N}_1\right)/\left({T}_2-{T}_1\right) $$

where *N*_1_ is the initial optical density measured at 650 nm, *N*_2_ is the final optical density measured at 650 nm, *T*_1_ is the initial time of cultivation, and *T*_2_ is the final time of cultivation.

Biomass doubling time (0–72 h) was calculated on the basis of the specific growth rate (Yadavalli et al. 2010).

Biomass yield is expressed in DCW gram per litre. Biomass productivity and compound productivity are expressed in DCW gram per litre per day.

The *P. kessleri* biomass was harvested by centrifugation after 12 days of cultivation and used for biochemical analysis.

### Lipid extraction

The determination of the oil content was carried out following a modified version of the Bligh and Dyer method (Bligh and Dyer 1959) described by Piasecka et al. (2014).

### Carbohydrate determination

Total simple sugars were estimated colourimetrically based on the anthrone method described by Trevelyan and Harrison (1952). The carbohydrate content of *P. kessleri* biomass can be measured by hydrolysing polysaccharides into simple sugars by acid hydrolysis and estimating the resultant monosaccharide. In hot acidic medium, glucose is dehydrated to hydroxymethyl furfural. This compound forms anthrone with a green-coloured product with an absorption maximum at 620 nm. The absorbance was measured at a wavelength of 620 nm, and the value of absorbance was compared with the glucose standard curve.

### Protein determination

The Kjeldahl method is a technique allowing measurement of the crude protein content in lyophilized material. Lyophilized microalgal biomass was subjected to digestion in sulphuric acid in the presence of a catalyst (Kjeltabs). Distillation was prepared in a distillation unit (Behr Labor-Technik GmbH). The amount of ammonia was determined by titration with a standard solution of 0.1 M HCl. The protein content of the lyophilized biomass of the microalgae was determined by the Kjeldahl method using the nitrogen-to-protein (N:P) conversion factor 5.95 (López et al. 2010).

### Analysis of fatty acids

The fatty acid analysis was performed by preparing fatty acid methyl esters (FAME) with BF_3_ in methanol and analysed by gas chromatography as described by Piasecka et al. (2014) and Krzemińska et al. (2015).

### Calorific value

The calorific value was determined using a semi-automatic bomb calorimeter (LECO AC600). The lyophilized algal biomass was combusted in the calorimeter, and benzoic acid was used as a calorific standard according to Oleszek et al. (2016).

### Statistical analyses

Each culture variant was performed in three biological replicates. Statistical analyses were carried out using STATISTICA 12 (StatSoft Inc., USA). To determine the effect of the feeding strategy on the productivities, growth parameters, and total oil, protein, carbohydrate, and FAME content, as well as the calorific value, two-factor analysis of variance was used; *p* values below 0.05 were considered significant, and next, the post hoc Tukey’s HSD test was used.

## Results

### Growth characteristics of *P. kessleri*

**Fig. 1:**
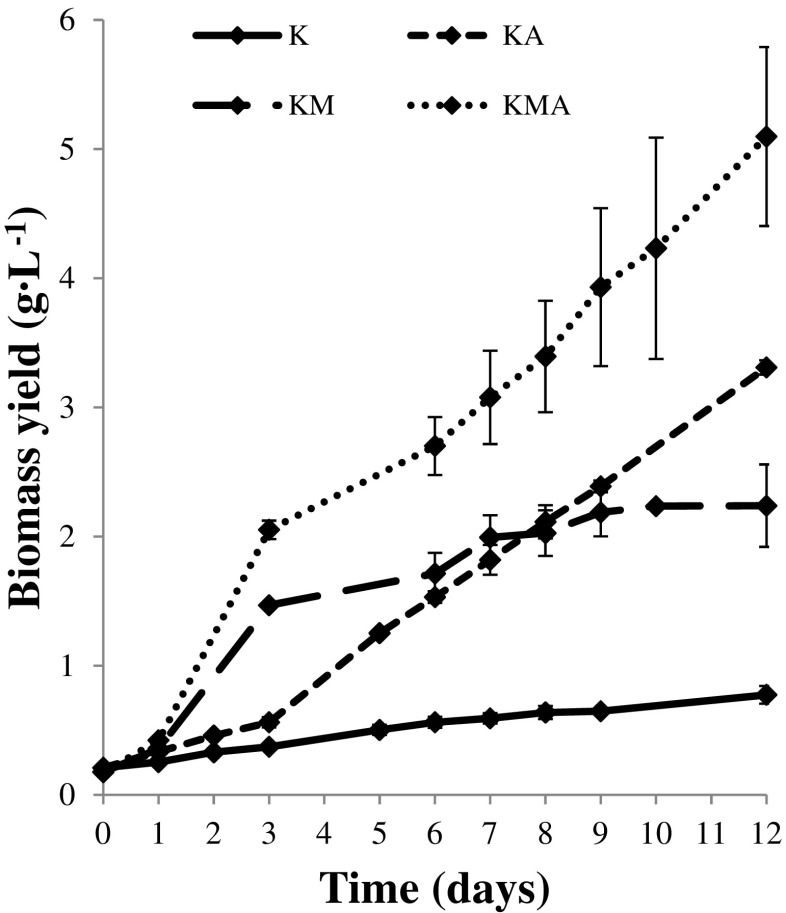
Growth curves of *Parachlorella kessleri* cultured under control (K), autotrophic (KA), photoheterotrophic (KM), and mixotrophic (KMA) culture conditions (the results are presented as the means of *n* = 9 measurements from three biological replicates; *error bars* represent standard deviation)

Figure 1 shows growth curves of *P. kessleri* for the control variant in the experiment (K) and three experimental variants: KA, KM, and KMA. The type of culture conditions significantly influenced microalgal growth and the length of the growth phase (Fig. 1). No obvious phases were observed in the control variant of the experiment (K culture). In the autotrophic culture conditions (KA), the growth curve showed a longer lag phase compared with photoheterotrophic (KM) and mixotrophic (KMA) cultures. Aeration in the KA culture allowed the algae to grow in the logarithmic growth phase, so no other phases were observed in the KA culture. In the KM culture, the growth curve showed three distinct phases of growth: the lag, log, and stationary phases with a noticeable slowdown. In the KMA culture with aeration, lag and log phases were observed. As in the case of the KA culture, no growth slowdown and stationary phase were noticed in the KMA culture conditions.

**Fig. 2:**
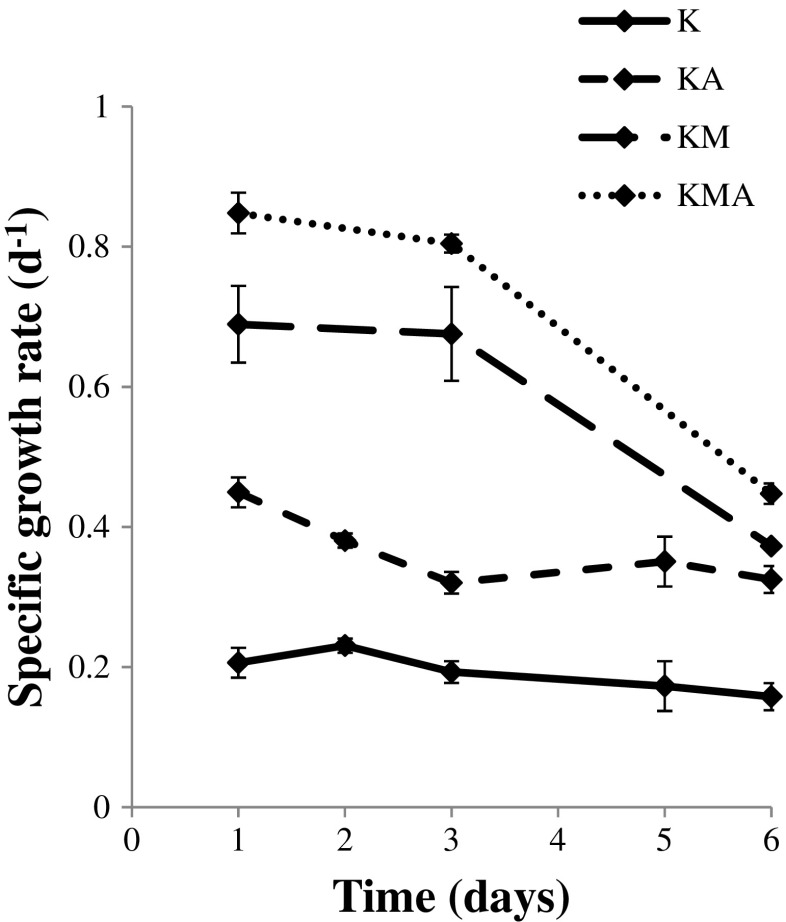
Dynamics of the specific growth rate during the *Parachlorella kessleri* cultivation (the results are presented as the means of *n* = 9 measurements from three biological replicates; *error bars* represent standard deviation)

The specific growth rate of *P. kessleri* for all feeding strategies is shown in Fig. 2. It was observed that during the first 3 days of cultivation, *P. kessleri* exhibited the highest specific growth rate. The specific growth rate exhibited a downward trend through the exponential growth phase.

The type of cultivation significantly influenced the biomass yield, biomass productivity, specific growth rate, and biomass doubling time (Table 1). The lowest biomass yield was observed in the K culture (0.77 g L^−1^). The autotrophic and photoheterotrophic cultivation of *P. kessleri* yielded fourfold and threefold higher biomass yield than in the control conditions, respectively. The mixotrophic cultivation with aeration guaranteed an almost sevenfold increase in the biomass yield of the *P. kessleri*. In the KMA culture of *P. kessleri*, after 12 days of cultivation, maximum biomass yield and biomass productivity reached 5.10 and 0.42 g L^−1^ day^−1^, respectively.

**Table 1:** Summary of growth parameters of *Parachlorella kessleri* cultured under control (K), autotrophic (KA), photoheterotrophic (KM), and mixotrophic (KMA) culture conditions

	Type of cultivation			
	K	KA	KM	KMA
Biomass yield (g L^−1^)	0.77 ± 0.07	3.31 ± 0.32	2.24 ± 0.05	5.10 ± 0.69
Biomass productivity (g L^−1^ day^−1^)	0.07 ± 0.01	0.28 ± 0.03	0.19 ± 0.00	0.42 ± 0.06
Specific growth rate 0–3 (day^−1^)	0.19 ± 0.00	0.32 ± 0.02	0.68 ± 0.07	0.80 ± 0.01
Biomass doubling time 0–72 (h)	86.2 ± 0.4	52.1 ± 2.6	24.9 ± 3.1	21.0 ± 0.3

Data are expressed as mean ± standard deviation of nine replicates

The growth parameters differed significantly depending on the conditions used for the cultivation (Table 1). The maximum specific growth rate (*μ* = 0.80 day^−1^) was obtained under the mixotrophic growth (KMA), and the minimum specific growth rate (*μ* = 0.19 day^−1^) was achieved under the control conditions (K). In addition, the biomass doubling time was shortened from 86 h in the control conditions to 21 h under the mixotrophic culture conditions.

### Biochemical composition of the biomass and productivity of cell components

**Fig. 3:**
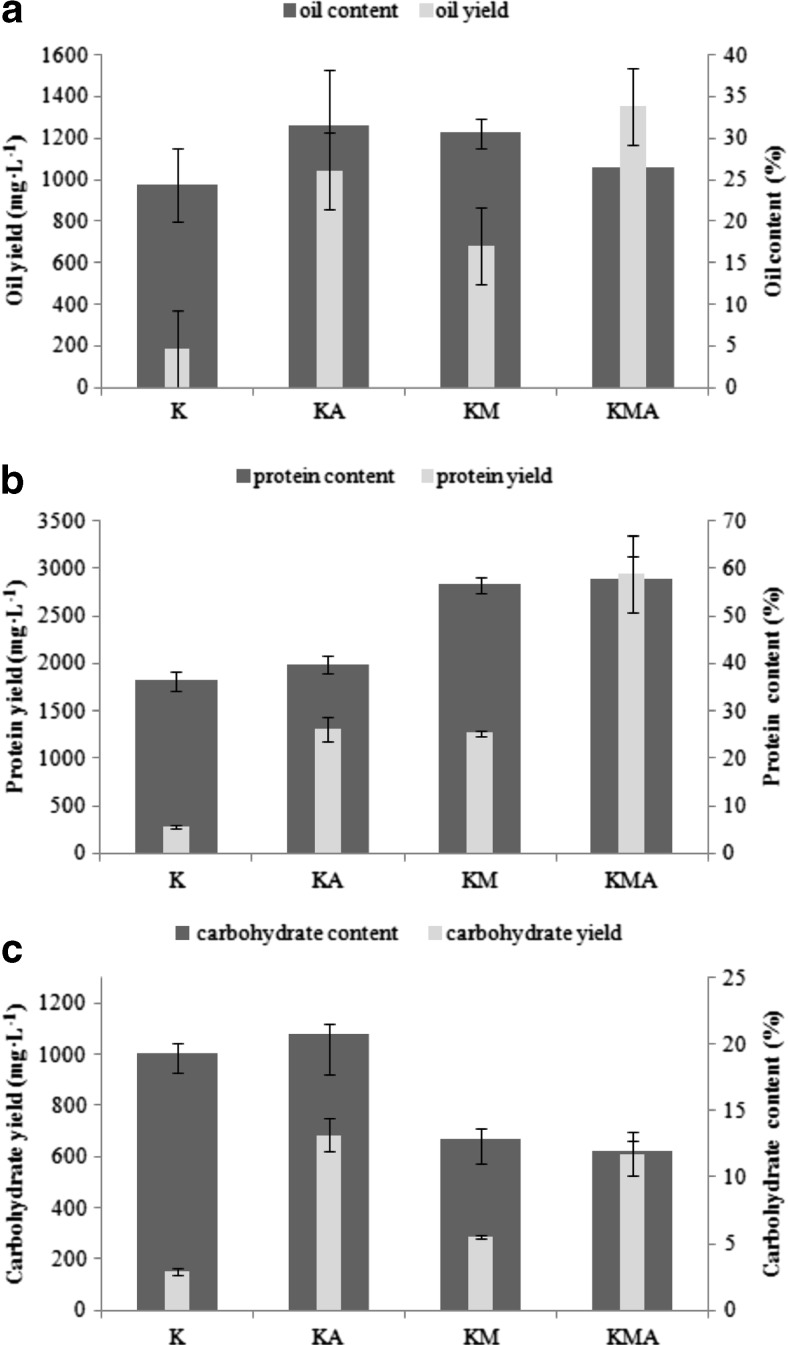
Effect of different feeding strategies on the biochemical composition and oil (**a**), protein (**b**), and carbohydrate productivity (**c**) (mg L^−1^) of *P. kessleri* (the results are presented as the means of *n* = 9 measurements from three biological replicates; *error bars* represent standard deviation)

The composition of *P. kessleri* biomass and biomass yield (g L^−1^) are summarized in Fig. 3. To enable a comparison with literature, our results also are converted to daily productivity of compounds in milligrams per litre per day (Table 2).

**Table 2:** Production of oil, carbohydrate, and protein expressed as daily productivity

	Type of cultivation			
	K	KA	KM	KMA
Daily oil productivity (mg L^−1^ day^−1^)	15.67 ± 1.39	86.71 ± 8.36	57.07 ± 1.40	112.56 ± 15.29
Daily carbohydrate productivity (mg L^−1^ day^−1^)	12.51 ± 1.11	57.09 ± 5.50	23.87 ± 0.59	50.97 ± 6.92
Daily protein productivity (mg L^−1^ day^−1^)	23.40 ± 2.08	109.62 ± 10.56	105.38 ± 2.59	244.95 ± 33.28

Data are expressed as mean ± standard deviation of nine replicates

Crude oil content and oil yield in *P. kessleri* biomass from K, KA, KM, and KMA conditions are presented in Fig. 3a. The maximum oil content (31.4%) was observed in autotrophic conditions. Photoheterotrophic cultivation with molasses and the mixotrophic cultivation with molasses and aeration yielded the total oil content of 30.6 and 26.4% dry cell weight, respectively. No significant difference (*p* > 0.05) was observed between accumulation of oil under KA, KM, and KMA conditions compared with the control conditions. Based on the biomass yield (g L^−1^) and the percentage of oil, the oil yield (mg L^−1^) was determined (Fig. 3a). Our findings showed that the oil productivity of *P. kessleri* ranged from a little as 15.67 mg L^−1^ day^−1^ for the control conditions to 112.56 mg L^−1^ day^−1^ for the KMA conditions.

The molasses-supplemented culture (KM) showed a significant increase in the protein content compared to the control conditions (Fig. 3b). The highest protein content (57.7%) was obtained in the KMA culture. In contrast, carbohydrate content decreased significantly from 19.4% (control conditions) to 12.8 and 12.0% for the KM and KMA cultures, respectively (Fig. 3c). The highest percentage of protein in *P. kessleri* biomass combined with the highest biomass yield resulted in the maximum protein productivity of 244.9 mg L^−1^ day^−1^ for the KMA conditions.

### Fatty acid profile with saturated, monounsaturated, and polyunsaturated fatty acid content

The fatty acid composition of *P. kessleri* from the control and each culture condition is summarized in Table 3. Lipids were mainly composed of C16 and C18 fatty acids under all testing conditions independent of the type of cultivation. C16 and C18 represented more than 80% of total fatty acids in the K and KA culture conditions and more than 70% of total fatty acids in the molasses-based culture conditions. Depending on the feeding strategy, *P. kessleri* cells displayed different values of the percentage of saturated fatty acids (SFA), monounsaturated fatty acids (MUFA), and polyunsaturated fatty acids (PUFA) as well as the percentage of individual fatty acids. The highest content of SFA (33.86%) was observed in the control conditions. The cultures grown on the molasses-based medium with aeration were rich in monounsaturated fatty acids (26.86%). The highest content of PUFA (42.83%) was in the autotrophic culture conditions. As shown in Table 3, in the control culture conditions, the percentages of palmitic acid (C16:0), linoleic acid (C18:2), oleic acid (C18:1), linolenic acid (C18:3), stearic acid (C18:0), and palmitoleic acid (C16:1) in total fatty acids were 23.38, 20.53, 17.81, 15.87, 10.48, and 1.1%, respectively. The level of C18:1 remained unchanged under all culture conditions. Under the KA culture conditions, it was observed that aeration did not alter the fatty acid profile significantly compared to the control. Molasses addition induced changes in the C16:1, C18:0, C18:2, and C18:3 content (*p* < 0.05), while molasses addition with aeration had a significant influence on changes in the C16:0, C18:0, and C18:2 content (*p* < 0.05). When molasses was supplemented, the content of palmitic acid, stearic acid, and linolenic acid decreased and this was accompanied with an increased content of palmitoleic acid and linoleic acid. When molasses with aeration was used, higher levels of palmitoleic acid and lower amounts of palmitic, stearic, linoleic, and linolenic acids were observed compared with the control conditions.

**Table 3:** Fatty acid profile of *Parachlorella kessleri* cultured under control (K), autotrophic (KA), photoheterotrophic (KM), and mixotrophic (KMA) culture conditions

FAME (%)	Type of cultivation			
	K	KA	KM	KMA
C16:0	23.38 ± 4.41	18.31 ± 0.97	17.01 ± 2.97	21.31 ± 1.42
C16:1	1.10 ± 0.00	1.03 ± 0.54	2.26 ± 0.33	2.79 ± 0.28
C18:0	10.48 ± 3.88	4.01 ± 0.55	3.19 ± 0.90	5.12 ± 1.12
C18:1	17.81 ± 4.40	17.51 ± 3.70	16.55 ± 1.28	17.52 ± 0.18
C18:2	20.53 ± 5.72	28.81 ± 1.48	23.25 ± 2.24	17.13 ± 0.52
C18:3	15.87 ± 5.37	14.02 ± 3.30	8.60 ± 0.96	7.05 ± 0.28
Total C16-C18	88.26 ± 5.07	83.51 ± 2.46	70.86 ± 3.01	70.92 ± 0.69
Others	11.74 ± 5.07	16.49 ± 2.46	29.14 ± 3.01	29.08 ± 0.69
SFA^a^	33.86 ± 6.65	22.32 ± 1.25	20.20 ± 3.48	26.43 ± 1.20
MUFA^b^	18.00 ± 4.29	18.36 ± 3.80	18.81 ± 1.55	26.86 ± 5.90
PUFA^c^	36.41 ± 4.15	42.83 ± 3.94	31.86 ± 3.00	24.17 ± 0.63

Data are expressed as mean ± standard deviation of six replicates

^a^Unsaturated fatty acids among total C16-C18

^b^Monounsaturated fatty acids among total C16-C18

^c^Polyunsaturated fatty acids among total C16-C18

### The calorific value of cell biomass

**Fig. 4:**
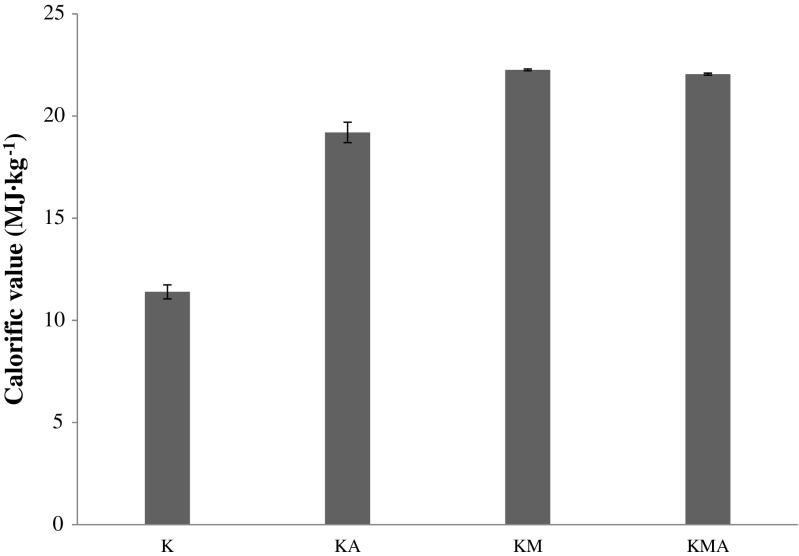
Calorific value of *P. kessleri* cultured under control (K), autotrophic (KA), photoheterotrophic (KM), and mixotrophic (KMA) culture conditions (the results are presented as the means of *n* = 9 measurements from three biological replicates; *error bars* represent standard deviation)

The calorific value of algal biomass was analysed for all four media (Fig. 4). The type of cultivation had a significant influence on the biomass calorific value (*p* < 0.05). The *P. kessleri* biomass was characterized by a relatively low calorific value (11.4 MJ kg^−1^) under the control conditions. The maximal calorific value (22.3 MJ kg^−1^) was achieved in the molasses-based medium, very similar to that of the KMA culture (22.1 MJ kg^−1^).

## Discussion

Growth of microalgae and the length of the growth phase depended on nutrient availability and depletion as well as formation of new cells in the culture medium. The lack of available nutrients required for algal growth in the culture medium, as in the control variant of the experiment, resulted in poor algal growth. Continuous supply of the substrate by aeration in both of KA and KMA cultures supported algal growth and caused absence of the stationary phase. In turn, the presence of an organic carbon source in KM and KMA shortened the lag phases. Organic carbon sources are metabolized quickly and provide instant energy to the microalgal cells that can be utilized for the growth (Dubey et al. 2015). The KM culture shows a typical pattern of growth for a batch culture.

Application of the molasses at a concentration less than 20 g L^−1^ seemed to be appropriate. During the photoheterotrophic cultivation with molasses (KM), the yield of *P. kessleri* biomass was 2.24 g L^−1^. Yeesang and Cheirsilp (2014) studied the effect of sugar cane molasses on *Botryococcus braunii*. In their experiment, they found that the optimal molasses concentration was 15 g L^−1^, which resulted in the highest biomass yield at day 15 of cultivation (3.05 g L^−1^). In their study, increased amounts of molasses up to 20 g L^−1^ decreased the biomass yield. The growth of *P. kessleri* in the medium containing molasses (KM) may be governed by the heterotrophic metabolism. This may be explained by ineffective light penetration during cultivation of microalgae on the molasses-based culture medium (Yeesang and Cheirsilp 2014). El-Sheekh et al. (2013) studied the effect of sugar cane molasses on *Scenedesmus obliquus* in similar environmental conditions (enrichment of Kessler medium with molasses; light intensity, 100 μmol photons m^−2^ s^−1^; temperature, 25 ± 1 °C). At 5 g L^−1^ molasses, they reported a biomass productivity of 0.292 g L^−1^ day^−1^. Molasses also can be used by other species of green algae. Yan et al. (2011), Liu et al. (2012), and Gautam et al. (2013) reported that molasses and molasses hydrolysate could support rapid growth of *Auxenochlorella protothecoides*, *Chlorella zofingiensis*, and *Chlorella minutissima*, respectively. The production of *P. kessleri* biomass was stimulated by molasses or aeration. Supplementation of the culture medium with molasses with simultaneous aeration caused maximal stimulation of growth. When simultaneously provided with light, CO_2_, and an organic carbon source in the mixotrophic culture conditions, the culture reached the maximum final biomass concentration (Yang et al. 2000). According to Villarejo et al. (1995), the presence of an organic carbon source in culture medium can change both photosynthesis and heterotrophic metabolism of *Chlorella* species. This could be explained by the observations of Marquez et al. (1993), who found that under growth with glucose in light, light and the organic carbon source fulfil the same role in the growth and cellular composition of microalgae. They reported that heterotrophic growth and photosynthesis might occur simultaneously and independently in *Spirulina* cultures. Our results indicate that the highest biomass productivity from *P. kessleri* could be achieved using molasses, a by-product of sugar refinery, as a carbon source and simultaneous aeration.

The type of nutrition causes changes in the kinetics of population growth of *P. kessleri* but does not modify the lipid content of cells. No difference in lipid content was observed between control and all variants of the experiment. Yeesang and Cheirsilp (2014) reported a 36.9% lipid content in cultivation of *B. braunii* at 15 g L^−1^ molasses concentration. To our knowledge, there is no published data on the use of molasses and simultaneous application of aeration and the influence of these culture conditions on the lipid content in any microalgal species. Our results suggested that the culture conditions applied were favourable and did not cause accumulation of lipids due to stress. Oil-rich green algae grown under optimal growth conditions exhibit an average total lipid content of 25.5% dry cell weight (Hu et al. 2008). Additionally, it is known that nitrogen-deficient media should be used during the cultivation in order to achieve lipid accumulation in the biomass of microorganisms (Ratledge 2004). It can be assumed that the molasses supplementation with/without aeration ensured beneficial environmental conditions not resulting in an increase in the oil yield due to the effect of the nitrogenous content of molasses. A critical parameter which determines the microalgal biomass suitability for production of algal-based biofuel is oil productivity (Griffiths and Harrison 2009). This parameter is strongly dependent on both biomass yield and oil content; however, a more dominant correlation was observed between lipid productivity and biomass productivity. Griffiths and Harrison (2009) reported that average lipid productivity for tested species from literature was 50 mg L^−1^ day^−1^. In their review, the authors reported that *Amphora*, *Neochloris oleoabundans*, and *Ankistrodesmus falcatus* were the most productive species. The oil productivity for these species ranged from 109 to 160 mg L^−1^ day^−1^. Our results highlight the potential of *P. kessleri* cultivated on molasses-based medium with aeration, which ensures high oil productivity; hence, *Parachlorella* is ranked among microalgal species with the highest oil productivity. It is of key importance for microalgal species selection for lipid production to present the oil content in relation to the specific growth rate and the biomass yield (Griffiths and Harrison 2009). *Parachlorella* *kessleri* characterized by rapid growth on molasses-based medium may exhibit greater lipid productivity than microalgae with a very high oil content.

Molasses is not only a source of sugar but it is also a rich source of nitrogen. The average amount of total nitrogen in beet molasses coming from national sugar factories is 1.8% (Polish Standard PN-R-64772, PN-ISO 6496:2002). Nitrogen is a very important constituent of protein synthesis and takes part in cell division and growth. During N deprivation, microalgal metabolism is redirected from proteosynthesis towards lipid and starch accumulation (storage compounds) (Adams et al. 2013; Procházková et al. 2014). In our experiment, N repletion occurred under molasses supplementation and yielded maximum protein accumulation in *P. kessleri* biomass, in both the KM and KMA types of culture conditions. Additionally, the presence of molasses in the culture media (KM and KMA cultures) caused a decrease in carbohydrate content. Griffiths (1965) found that molasses stimulated the protein production in the cultivation of *Scenedesmus*. Our results showed that the use of molasses-supplemented medium with/without simultaneous aeration allowed obtaining two valuable components (oil and protein) with high productivities from the biomass. Microalgal biomass has the potential to be an important and sustainable renewable energy feedstock for biodiesel production. A more suitable and economically feasible process of microalgal lipid production can be achieved by combining lipid production with other applications. In order to develop a more sustainable process, all compounds (lipids, proteins, and carbohydrates) originating from the microalgal biomass should be used (Wijjfels et al. 2010; Bellou et al. 2014). *Parachlorella kessleri* biomass grown in molasses-based medium may find application not only in biofuel production but also in human and animal nutrition (feed additives), cosmetics, organic farming (fertilizers), and pharmaceuticals because of its high protein content and high biomass and protein productivity. In addition, the use of the low-cost agricultural by-product in mixotrophic and photoheterotrophic cultures of microalgae is of great importance especially in reducing the cost of biomass production.

In the Chlorophyceae, the major fatty acids are palmitic acid (C16:0) and oleic acid (18:1). The polyunsaturated fatty acids include linoleic acid (C18:2) and linolenic (C18:3). In general, saturated and monounsaturated fatty acids are predominant in most algae examined in the literature (Hu et al. 2008). Wang et al. (2013) found that *Chlorella kessleri* biomass under mixotrophic culture conditions accumulated mostly C16 and C18, which represented more than 95% of total FAME. The amount of FAME was always higher for low nitrogen and phosphate concentration cultures compared to medium with high nutrient concentrations (Lin and Lin 2011). The composition and structure of fatty acid methyl esters determine the most important properties of biodiesel. The results in this paper show that the fatty acid composition is similar to that of diesel fuel, mainly consisting of long-chain fatty acids (C16 and C18), which are most preferable for biodiesel fuel. However, a higher content of total FAME ensures hydrating and stabilizing properties, which improve the quality of biodiesel (Knothe 2005, 2013). The addition of molasses to the culture medium changed the yield and composition of FAME. Generally, the photoheterotrophic and mixotrophic cultures of *P. kessleri* contained lower contents of polyunsaturated fatty acids, which is recommended for biodiesel. Biodiesel produced from biomass enriched with polyunsaturated fatty acids tends to have instability problems during storage (Hu et al. 2008). When molasses was supplemented to the culture media, low linolenic acid content was observed. The total content of C18:3 decreased from 15.87% (control conditions) to 8.60 and 7.05%, respectively, for the KM and KMA cultures. These results met high quality standards complying with the specification requirements of EN 14214. Similar results were obtained by Wang et al. (2013), who also indicated mixotrophic *C. kessleri* growing under different carbon sources as a future industrial biodiesel producer.

The calorific value is a parameter that determines the energy potential of cell biomass. For the KM and KMA cultures, the *P. kessleri* biomass achieved maximal calorific value. The main contribution to the calorific value of microalgal cells is derived from first of all their lipid (9.4 kcal), next protein (5.65 kcal), and finally carbohydrate (4.2 kcal) content (Cleveland and Morris 2013). A calorific value between 18 and 21 kJ g^−1^ is characteristic for microalgae with the total oil content of 20–30% grown under normal conditions (Scragg et al. 2002). Summarizing, the calorific value is another parameter beyond the lipid and protein content that indicates KM and KMA culture conditions as optimal for *P. kessleri* cultivation.

In conclusion, the supplementation of the medium with beet molasses as an organic carbon source in the photoheterotrophic and mixotrophic cultivation significantly affected *P. kessleri* biomass growth, oil and protein productivity, fatty acid profile, and calorific value. The results showed that application of molasses makes *P. kessleri* cultivation economically favourable given the use of a cheap carbon source replacing glucose. Our study highlights the potential of algal biomass, especially in production of oils for biodiesel in combination with production of other metabolic compounds having application in various bioeconomy sectors.
